# Supplementary material for: Research on autonomous obstacle avoidance of mountainous tractors based on semantic neural network and laser SLAM
Source: PLoS One. 2025 May 21;20(5):e0323631. doi: 10.1371/journal.pone.0323631 (PMC12094762; doi:10.1371/journal.pone.0323631)
Supplement: Attachment — Submitted filename: Research on Autonomou.docx [file pone.0323631.s002.docx]

**Article title**：Research on Autonomous Obstacle Avoidance of Mountainous Tractors Based on Semantic Neural Network and Laser SLAM

**Academic comments on the article**

This paper proposes an autonomous obstacle avoidance method for mountain tractors based on semantic neural networks and laser SLAM, but it has significant flaws. The research motivation fails to clarify the unique challenges of mountainous versus farmland environments. Technical details (e.g., point cloud registration in SLAM, neural network pruning strategies) lack mathematical validation and quantitative analysis. Experimental comparisons are limited to traditional algorithms (GET/FGM), omitting mainstream methods (e.g., RRT* or reinforcement learning). Critical aspects such as dynamic obstacle testing, sensor fusion, energy consumption, and safety evaluation (e.g., ISO 25119 metrics) are inadequately addressed. Data availability is unclear, references are outdated, and recent GEITS studies (e.g., spatiotemporal A* optimization, non-uniform quadtree mapping) are not cited. Practical deployment feasibility (e.g., adverse weather impacts) is ignored. Major revisions—including technical refinement, expanded experiments, mandatory GEITS citations, and public data/code—are required to avoid rejection.

**Comments**

1. The introduction does not clearly articulate the unique challenges of autonomous obstacle avoidance for mountain tractors. A detailed scenario analysis is needed to highlight the differences from traditional farmland environments.
2. The mathematical derivation of plane feature matching in Equations (8)-(10) is overly simplified, with no explanation of how noise in point cloud registration is addressed. It is recommended to refer to the GEITS paper "A non-uniform quadtree map building method including dead-end semantics extraction" (DOI:10.1016/j.geits.2023.100071) for map construction methods.
3. Only GET and FGM algorithms are compared, without including mainstream methods like RRT* or deep reinforcement learning. It is recommended to reference the baseline methods in the GEITS paper "A review on reinforcement learning-based highway autonomous vehicle control" (DOI:10.1016/j.geits.2024.100156).
4. The coordination mechanism between A* and DWA is vaguely described, with no explanation of how global paths are dynamically updated to adapt to environmental changes. It is recommended to refer to the spatiotemporal constraint optimization methods in the GEITS paper "Spatiotemporal-restricted A∗ algorithm as a support for lane-free traffic" (DOI:10.1016/j.geits.2024.100159).
5. Key metrics such as inference speed (FPS) and memory usage of the lightweight network are missing, making it impossible to prove its suitability for embedded devices.
6. The data availability statement claims "data is within the manuscript," but no point cloud dataset or code links are provided, violating reproducibility principles. Data should be uploaded to a public platform.
7. The system's power consumption and its impact on tractor endurance, especially in mountainous areas without charging facilities, are not discussed. This issue is critical and should be addressed.
8. Safety is only measured by "comfort score," without introducing quantitative metrics from agricultural machinery safety standards (e.g., ISO 25119), such as minimum obstacle avoidance distance or emergency braking response time.
9. In distributed control scenarios, communication delays between LiDAR and computing units may affect real-time performance. Delay tolerance testing should be added.
10. Some references (e.g., [9] on laser SLAM) are from before 2023 and you can cite the latest GEITS paper "Deep transfer learning for intelligent vehicle perception: A survey" (DOI:10.1016/j.geits.2023.100125) on transfer learning methods

Restructure the methodology section to clarify technical details; Expand the experimental section (dynamic scenarios, multi-sensor comparisons); Add feasibility analysis for practical deployment.
